# Endoscopic treatment of ectopic teeth in the maxillary sinus

**DOI:** 10.4317/jced.57905

**Published:** 2021-03-01

**Authors:** Muhamed Masalha, Shay Schneider, Firas Kassem, Ilan Koren, Ron Eliashar, Ariel Margulis, Roee Landsberg

**Affiliations:** 1 Department of Otolaryngology-Head and Neck Surgery, Emek Medical Center in Afula, The Ruth and Bruce Rappaport School of Medicine, The Technion Institute of Technology in Haifa, Israel; 2 A.R.M, Center of Otolaryngology-Head and Neck Surgery. Assuta Medical Center in Tel Aviv, Tel Aviv University, Israel; 3 Department of Otolaryngology-Head and Neck Surgery, Meir Medical Center in Kfar Saba, Sackler school of Medicine, Tel Aviv University, Tel Aviv, Israel; 4 Department of Otolaryngology- Head and Neck Surgery, Rabin medical center in Petah Tikva, Tel Aviv University, Israel; 5 Department of Otolaryngology- Head and Neck Surgery, Hadassah Medical Center, and the Faculty of Health Sciences, Jerusalem Hebrew University, Jerusalem, Israel

## Abstract

**Background:**

Ectopic teeth in maxillary sinus is rare and are usually removed via sub-labial trans-canine fossa approach (SLCFA). The aim of our study was to present our experience with extraction of ectopic teeth in the maxillary sinus via transnasal endoscopic approach (TEA).

**Material and Methods:**

Rhinologists were asked to share their experience in the management of ectopic teeth in the maxillary sinus. Data were reviewed retrospectively.

**Results:**

Eleven cases were reported in 10 patients from 2010 to 2019, six males and four females with a mean age of 33.5 +/-17 years (range 16 to 61). Seven patients complained of sinonasal symptoms, two were diagnosed incidentally during routine dental work-up, and one had oro-antral fistula. In eight patients, a cyst coexisted within the maxillary sinus. Teeth were located arbitrarily within the sinus. All cases were operated by TEA. One patient had self-limited periorbital emphysema, one had transient cheek numbness, and one had early post-operative bleeding that stopped after endoscopic cauterization. Long-term follow-up revealed good clinical outcomes.

**Conclusions:**

Transnasal endoscopic removal of ectopic teeth from the maxillary sinus is a feasible and rational approach when SLCFA is contraindicated.

** Key words:**Ectopic teeth, dentigerous cyst, endoscopic sinus surgery, Caldwell-Luc.

## Introduction

Tooth embryogenesis and development is a complex and multistep synergistic process of the oral epithelium and basal mesenchymal tissue (1). Development of ectopic teeth occurs when this synergy is disrupted. Three known processes are responsible: developmental disturbance (e.g. cleft palate), iatrogenic actions (e.g. trauma), or a pathologic process (e.g. tumor or cyst).

Ectopic tooth eruption in the dentate region is widely discussed in the literature, occurring most often in the mandible, and among females. Incisors, canines, and premolars are the most commonly affected sites. Ectopic eruption in non-dentate regions such as the maxillary sinus is very rare. Ectopic maxillary teeth typically arise from the third molar, and most are asymptomatic. However, review of the literature reveals that ectopic tooth eruption may be associated with a variety of clinical manifestations, including facial pain, unilateral purulent nasal discharge, headache, nasal obstruction, facial edema, chronic sinusitis, epiphora, and numbness (2-4). The literature on ectopic maxillary teeth is mostly limited to case reports (5), describing a variety of presentations, including nasolacrimal duct obstruction (6-7), ectopic teeth close to a migrated dental implant in the maxillary sinus (8), and even a maxillary ectopic tooth leading to elevation of the orbital floor (7). Several locations of ectopic teeth within the maxillary sinus, with varying pathologies, have been described (5). Usually diagnosis is an incidental finding during routine dental or sinonasal imaging (6). Ectopic teeth in the maxillary sinus are radio-opaque and are thus easily diagnosed on radiography (9). In some cases, further imaging modalities such as CT are required to localize the ectopic tooth and plan treatment (10).

Ectopic teeth in the maxillary sinus are usually resected using a SLCFA, with or without using a sinuscope (9). However, in cases such as Pathological bone quality, dentofacial deformities, and Severe bone atrophy, oral surgeons may be less enthusiastic to use the trans-oral sublabial approach. In recent years, however, ectopic teeth in the maxillary sinus have been removed endoscopically via the nasal route (8).

In this study, we share our experience in the endoscopic surgical management of ectopic teeth in the maxillary sinus. We offer this technique as an alternative when the dentists wish to avoid the SLCFA. Most reports dealt with a very small number of patients, and to our knowledge, this is the largest series of patients in which removal was accomplished by this technique. We also review the literature in an effort to establish a better understanding of this disorder, treated by both Dentists and Otolaryngologists.

Material and methods

-Study population

Members of the Rhinologic Society were asked to share their experience in evaluation and management of ectopic maxillary teeth. The medical charts of all patients diagnosed and surgically treated at 6 different medical centers for ectopic tooth in the maxillary sinus from January 2010 to June 2019 were reviewed. Data were collected regarding patients’ age, gender, symptoms, imaging findings, location of the ectopic teeth, indications, surgical indications and approach, complications and outcome.

-Surgical procedure

Endoscopic procedures were performed via the nasal route, which enables entry to the maxillary sinus either via middle meatal antrostomy (MMA), using the natural opening of the sinus, or by inferior meatal antrostomy (IMA), creating a new opening beneath the inferior turbinate, or by endoscopic medial maxillectomy (EMM) in which most of the medial maxillary wall was removed. For MMA, a 4mm Hopkins telescope with different vision angles was utilized. The uncinate process was resected to expose the natural sinus opening, which was then further widened in order to facilitate entry into the sinus and the use of blunt instruments. IMA was performed using the same route and telescope. Antrostomy was usually established about 0.5cm posterior to Hasner’s valve and then widened anteriorly and posteriorly. Ectopic teeth were usually loose, and their extraction was accomplished by grasping angled Blakesley. Sometimes, prior manipulation of the teeth with a Sinus Seeker was necessary. EMM was performed by excision of the inferior turbinate and widening the MMA to the maxillary sinus floor inferiorly, the lacrimal sac anteriorly and the pterygoid plate posteriorly.

-Statistical analysis

Mean, median and standard deviation were computed for the continuous data and percent were computed for the categorical data.

## Results

Ten patients (6 females and 4 males) with 11 ectopic teeth and with a mean age of 33.5 +/-17 years (range 16 to 61) were included in the study.

Of these 10 patients, 7 patients (73%) had been referred to Rhinology consultation due to sinonasal symptoms suggesting recurrent maxillary sinusitis, such as nasal discharge, facial pain, facial numbness, and nasal stuffiness or obstruction. Two patients (18%) were diagnosed incidentally during routine dental work-up and were referred for Rhinologic treatment; Of these 2 patients, 1 was asymptomatic and the other had mild symptoms. The last patient (9%) had chronic oral purulent discharge that started after periodontal abscess incision and drainage and was diagnosed with oro-antral fistula. One patient (9%) had bilateral ectopic tooth. Patient details are summarized in Table 1.

**Table 1:**
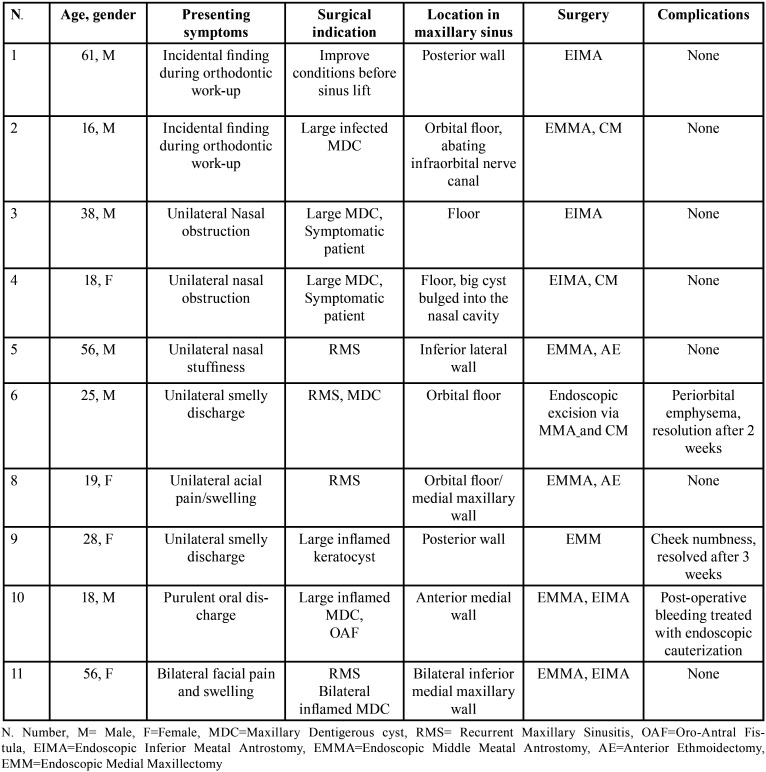
Patient summary.

**Figure 1:**
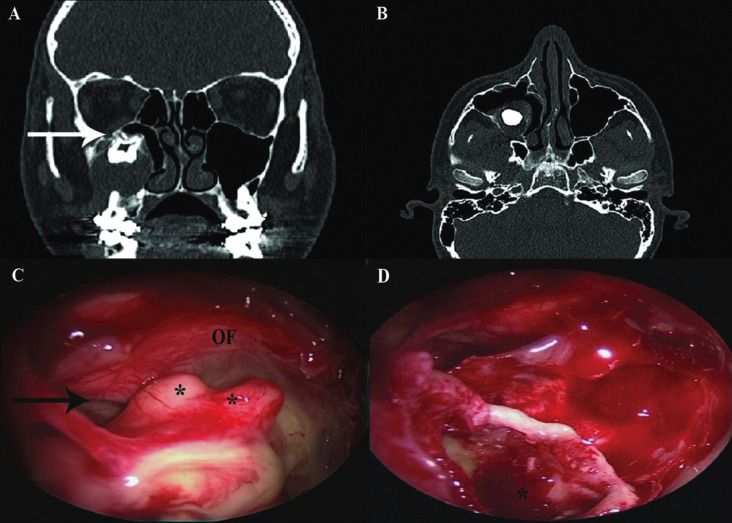
(A, B) Non-contrast CT scan of the maxillary sinus in a 16-year-old male (patient 2). (A) Coronal view. The ectopic tooth sits on top of a large cyst, which erodes the alveolar crest and the lateral maxillary sinus wall. The tooth abuts the orbital floor and the infraorbital nerve canal (arrow). (B) Axial view. (C, D) Intraoperative endoscopic images in the same patient. (C) Following wide middle antrostomy, the dentigerous cyst comes into view. Note orbital floor (OF), infraorbital nerve canal (arrow), tooth roots (asterisks). (D) Maxillary sinus cavity following cyst marsupialization. The bottom of the cyst (asterisk) is seen.

All patients underwent preoperative CT and endoscopic nasal evaluation. Location of the ectopic teeth, defined by the preoperative CT scan and intraoperatively, varied: one was located in the wall of the lateral maxillary sinus, two abutted the orbital floor, two were within the floor of the maxillary sinus, two in the posterior wall of the maxillary sinus, three in the medial wall and one at the junction of the orbital floor and the medial wall of the maxillary sinus. In seven cases, a dentigerous cyst coexisted with the ectopic tooth within the maxillary sinus (Fig. 1). In one case, an odontogenic keratocyst was associated with the ectopic tooth.

**Figure 2:**
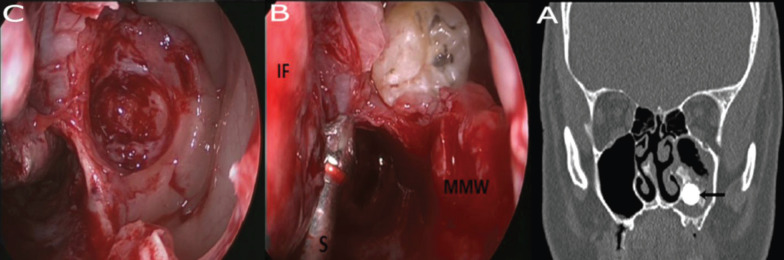
(A) Non-contrast CT, coronal view, of the sinus in a 61-year-old male (patient 1). Ectopic tooth (arrow) in the maxillary sinus wall, surrounded by bony formations. The sinus is partially inflamed. (B, C) Endoscopic images in the same patient. (B) Trans-nasal inferior antrostomy exposes the ectopic tooth in the sinus cavity. Note the inferior turbinate (IF), suction cannula (S), and medial maxillary wall (MMW). (C) Following tooth extraction, the aberrant tooth socket is at the posterior maxillary sinus wall.

Eleven teeth (100%) were treated endoscopically via the transnasal approach. The approach was performed through MMA in 4/11cases, and through IMA, sparing the ostiomeatal complex, in 3/11 cases (Fig. 2) and 3/11 teeth were extracted through combined MMA and IMA. One ectopic tooth required EMM.

Follow-up ranged from 3 months to 2 years. In all patients’ symptoms resolved and endoscopy indicated normal maxillary sinus mucosa. The symptoms of the patient with oro-antral fistula completely resolved after surgery and the fistula was closed without any further intervention. Postoperative complications occurred in three cases; one developed mild periorbital emphysema which resolved spontaneously with no orbital compromise, one had transient cheek numbness and one had post-operative nasal bleeding several hours after surgery and was treated with endoscopic cauterization in the operating room (Table 1).

## Discussion

The eruption of ectopic teeth in the dentate region is a well-known phenomenon often seen in dentistry clinical practice (11). Eruption of ectopic teeth in a non-dentate area is very rare; however, cases of ectopic teeth have been reported in the nasal cavity, nasal septum, maxillary antrum, orbit, chin, mandibular condyle, and coronoid process (12). Due to its rarity, the literature on tooth impaction in the maxillary sinus is limited to case reports. Three previous studies reviewed the cases published in English literature and reported on only 35 cases of ectopic teeth in the maxillary sinus from 1927–2011, and more recently on 65 cases from 1966–2012 (13-14).

During the last two decades, endoscopic sinus surgery (ESS) has become the mainstay of surgical treatment of sinonasal pathologies. Angled endoscopes and curved instrumentation are used to access hidden and remote corners in the paranasal sinuses. As such, it is reasonable to apply this approach when removing ectopic teeth in the maxillary sinus, as an alternative to the traditional SLCFA, when the latter is not recommended.

Levin *et al.* (15) conducted a systematic review on all 27 cases of endoscopic removal of ectopic sinonasal teeth in the literature. the review demonstrates that there is scarce literature on endoscopic removal of maxillary sinus and nasal cavity teeth removal but provide insight into the utility of endoscopic surgery as a safe and effective management strategy.

In our series, endoscopic removal of ectopic sinonasal teeth via a trans-nasal approach was used safely and effectively in 11/11 cases (100%).

Ectopic teeth have been reported in a variety of locations within the maxillary sinus antrum. Jude *et al.* and Hasbini *et al.* (5) reported on two medially located ectopic teeth in the maxillary sinus obstructing the sinus. The first was located inferiorly and was removed via a sub-labial anterior antrostomy. In the second case, the tooth was removed endoscopically through the nasal route due to its higher location at the level of the ostiomeatal complex. The authors suggested the endoscopic TEA whenever the tooth is situated medially and high. For posteriorly or laterally located teeth they advised a combined endoscopic trans-nasal and sub-labial approach, with trocar insertion (5).

This location variability is well shown in our series, in which the teeth were found to be anchored, arbitrarily, on any of the sinus walls. All were removed endoscopically with minimal morbidity and good outcomes. Resection of an ectopic tooth is a single foci problem, which makes the specificity and accuracy of the endoscope and related surgical instruments an appropriate surgical choice. In all cases, we could extract the ectopic teeth from the maxillary sinus, including cases where they were located medially, laterally, posteriorly, or even abutting the orbital floor. Entry to the maxillary sinus can be achieved through MMA or IMA, depending on the tooth’s location. Ectopic teeth located in the upper parts of the maxillary sinus may be best approached via MMA, while those located posteriorly or inferiorly in the maxillary sinus may be better accessed via IMA, without violating the osteomeatal complex. In EMM the inferior turbinate is usually scarified which may cause adverse effects such as empty nose syndrome (16). Only in one case in our series EMM was required without reported complications.

Ectopic teeth can be firm, smooth and large. To facilitate endoscopic tooth extraction, we suggest using a long angulated grasping instrument. Sometimes, when the nasal anterior opening is too narrow, removing the teeth in a retrograde fashion through the nasopharynx and oral cavity is required.

The sub-labial approach is associated with some adverse effects such as facial swelling, infraorbital nerve injury, pain and/or numbness of the teeth/gums, epistaxis, oro-antral fistulae, epiphora, and dental discoloration (5,17,18). In our series, self-limited periorbital emphysema occurred in one patient (1/10, 10%), transient facial numbness in one patient (1/10, 10%) and epistaxis in one patient (1/10, 10%). Seven patients (7/10, 70%) experienced no adverse effects or complications. At long-term follow-up, patients experienced complete resolution of their symptoms. Although the SCFLA is a direct and easy approach to the maxillary sinus with relatively low comorbidities, the TEA approach was found to be feasible and safe, and should be considered when the trans-labial approach is contra-indicated or when the dentist desire to avoids violation of the oral anatomy for future maxillary dental implantation with no previous intervention, or when associated sinonasal disease coexists and require endoscopic treatment anyway.

A coexisting dentigerous cyst accompanying the ectopic tooth in the maxillary sinus was reported in several articles (3,11,14,17,19-21). This cystic correlation is consistent with our experience, where a dentigerous cyst associated with the ectopic tooth was found in 7/11 (63.6%) of the ectopic teeth. And in one patient another cystic formation, keratocyt, was found. Dentigerous cysts associated with ectopic tooth in maxillary sinus are fairly rare, with only 17 cases reported between 1980 and 2011 (3). These epithelial cell-lined cysts may displace the embryonic tooth bud into an ectopic position such as the maxillary sinus (21). The cysts have the potential for malignant transformation to carcinoma or ameloblastoma, which may arise from either the epithelial lining or the remnant of odontogenic epithelium in the cyst wall. When an ectopic tooth in the maxillary sinus is associated with a cystic lesion, surgical extraction of the tooth along with cyst enucleation is clearly indicated (17).

Lai *et al* postulated that there may be a higher incidence of maxillary sinus ectopic teeth in men than in women (14). In our study, 6/10 (60%) were men, this male predominance among our patients is consistent with Lai *et al.* results.

Most patients with ectopic tooth in the maxillary sinus are asymptomatic; therefore, they may go unnoticed throughout life, or be diagnosed incidentally during dental or other skull workup. Symptomatic patients may present with chronic or recurrent sinusitis, nasal polyps, facial swelling, maxillary discomfort, and rarely, epistaxis and nasolacrimal duct obstruction (11). Asymptomatic patients may be followed-up periodically without immediate intervention. Surgical extraction may be warranted due to evolving symptoms, an associated dentigerous cyst, or plans for dental treatment or surgery in the sinus area. Only 1/10 patients in the current series was completely asymptomatic, another had very mild symptoms. Both were diagnosed incidentally. The other eight patients presented with symptoms suggestive of chronic maxillary sinusitis or oro-antral fistula after dental treatment. This is not surprising, assuming that patients presenting with sinonasal symptoms would seek for an Otolaryngologist.

For ectopic teeth in the maxillary sinus, plain radiograph is often diagnostic; however, when associated with sinusitis and an opaque sinus, it may be difficult to distinguish the tooth from reactive sinusitis with Water’s view radiographs (11). In such cases, panoramic radiograph is a cost-effective study providing sufficient information for diagnosis and intervention since it can demonstrate the highly radio-opaque tooth and surrounding soft tissue reaction (21). However, CT is the gold standard modality for evaluation of the sinonasal region. CT scan of all sinuses, with 3D reformation, can be of great benefit in delineating ectopic tooth morphology, sinus pathology, tooth localization, and adjacent sinus involvement, as well as surgical planning. Moreover, CT is useful in differentiating ectopic tooth from other pathologies presenting as radiopaque lesions within the sinus, such as a rhinolith, tumors with calcifications, a dermoid cyst, and bacterial or fungal infection. A structure with density equivalent to a tooth with a central cavity, located inside the maxillary sinus, suggests the diagnosis, although ectopic teeth may sometimes be of anomalous morphology compared with the typical normal dentition (11). Since the nasal route was our preferred approach for surgical intervention, all of our patients were evaluated with preoperative CT and endoscopic nasal examination. Meticulous review of the preoperative CT is highly advised, with special attention to the location and integrity of maxillary sinus walls, size and shape of the teeth, the passage through the nasal cavity, associated cysts, and adjacent vital structures. This preparation allowed us to develop a precise surgical plan.

In conclusion, endoscopic surgery via the transnasal approach represents a feasible and safe strategy for the removal of ectopic teeth in the maxillary sinus. This method led to an excellent outcome. Sparing the oral cavity enables future maxillary dental implantation or sinus lift in an anatomical field that has not been violated by a previous surgery. We recommend consultation with both an oral surgeon and a rhinologist when removal of a maxillary sinus ectopic tooth is considered.
